# Burnout Risk and Protection Factors in Certified Nursing Aides

**DOI:** 10.3390/ijerph15061116

**Published:** 2018-05-30

**Authors:** María del Mar Molero Jurado, María del Carmen Pérez-Fuentes, José Jesús Gázquez Gázquez Linares, María del Mar Simón Márquez, África Martos Martínez

**Affiliations:** 1 Department of Psychology, University of Almería, Almería 04120, Spain; mmj130@ual.es (M.d.M.M.J.); sej473@ual.es (M.d.M.S.M.); amm521@ual.es (Á.M.M.); 2 Department of Psychology, Universidad Autónoma de Chile, Región Metropolitana, Providencia 7500000, Chile; jlinares@ual.es

**Keywords:** burnout, risks, protective factors, nursing

## Abstract

Studies have found a higher risk of burnout among employees in the healthcare sector. As such, this study focused on Certified Nursing Aides (CNAs) who have shown a high prevalence of burnout and are therefore considered an especially vulnerable group. The objective of this study was to identify the relationships between some organizational, personal, and sociodemographic factors and burnout. The final study sample included 278 working CNAs with a mean age of 40.88 (SD = 9.41). To compile the data, an ad hoc questionnaire was used to collect sociodemographic information. To collect professional and employment information, we used the Brief Emotional Intelligence Inventory for Adults, the Brief Questionnaire on Perceived Social Support, and the General Self-Efficacy Scale. The results showed that Burnout Syndrome is significantly and negatively related to all the emotional intelligence factors, self-efficacy, and perceived social support. The risk of burnout is higher in younger persons and in permanently employed professionals. General self-efficacy and stress management act as protective factors against the likelihood of burnout. This study suggests that organizations should urge coaching and transformational leadership training programs to promote the wellbeing and organizational commitment of workers.

## 1. Introduction

Burnout has been widely studied in the academic and professional fields. The World Health Organization (WHO) considers burnout syndrome an occupational disease that can affect workers in many occupational sectors [1], being prevalent in 13–27% of the active population [2]. The literature reviewed shows that employees in the healthcare sector are at a higher risk of this syndrome [3]. Therefore, we focused on Certified Nursing Aides (CNAs), who have a reported 26–50% prevalence of burnout and are therefore considered an especially vulnerable group [4].

In general, burnout syndrome is characterized by (1) gradual physical and mental exhaustion, (2) feelings of cynicism, detachment, and negative attitudes toward the job, and (3) a decrease in professional efficacy resulting from the work context [5]. The literature also emphasizes both its organizational (job performance and absenteeism) and health consequences to workers. Burnout has been related to various psychological problems, such as depression, anxiety, and mood disorders, and also physical problems, including musculoskeletal and cardiovascular problems, Type 2 Diabetes, sleep disorders, headache, and respiratory and gastrointestinal infections [6].

Empirical research on burnout has a crucial reference milestone in the studies by Maslach [7,8]. through the introduction of the Maslach Burnout Inventory (MBI) [9], and since, various adaptations and new evaluation models have been developed, such as the Cuestionario Breve de Burnout (Brief Burnout Questionnaire; BBQ) [10,11] as a reference.

At the beginning of the 21st century, a new theoretical model was developed: the Job Demands-Resources Model (JD-R) [12]. This model provides an improved understanding of the phenomenon and enables predictions of wellbeing and performance in the job [13]. This model identifies work demands and resources as possible antecedents of burnout, in which two categories trigger different processes: deterioration of employee health and motivational processes [12,13]. The model has also identified personal resources of the workers as relevant because they are positively related with engagement and performance while buffering the negative impact of job demands [12].

Special attention has focused on healthcare professionals in empirical burnout. Most studies have used occupational samples in the scope of healthcare [14,15,16]; however, only a few studies have been concerned with the work context in which nursing aides perform their work [4,17,18]. Thus, empirical studies have been directed at identifying the antecedents that have a close relationship with burnout, emphasizing heavy workload [17], time in the job, work shifts [4], employment situation, repeated exposure to traumatic events [19], role conflict and ambiguity [20], perceived social support [21], permanent contracts and long-term [22,23], strategies for coping [24,25], and job autonomy [4].

Personality traits or characteristics that buffer the negative effect of job demands and act as protection factors against job stress have also been identified [17]. The literature has underlined the importance of Emotional Intelligence (EI), understood as skills for understanding, perceiving, and adaptive management of one’s own emotions and those of others, and their relationship with engagement and job performance [26]. Except for “Neuroticism”, the other four wide personality traits have been demonstrated to be positively correlated with EI and engagement [27]. Similarly, the importance of workers’ perceived self-efficacy with regard to their ability to control their surroundings has been reported in the literature as a burnout protection factor and predictor of engagement [14,28]. Empirical studies also have included sociodemographic variables as possible predictors of burnout, emphasizing age [19], sex [17], and marital status of workers [4].

Our main objective was to identify the relationships between some organizational, personal, and sociodemographic factors and burnout in a sample of Spanish CNAs. Despite the innumerable studies published in this area, one of the strengths of our study is the interest in the wellbeing of CNAs in hospital contexts, which has been infrequently undertaken in the literature. It thus provides better comprehension of the phenomenon that could lead to the design of future preventive intervention.

## 2. Materials and Methods

### 2.1. Participants

The original sample included 374 Certified Nursing Aides (CNAs) in Andalucía, Spain randomly selected from different health centers who were actively employed at the time data were collected. The final study sample included 278 participants, of whom 71.6% (*n* = 199) were temporary and 28.4% had permanent contracts.

The mean age of the participants was 40.88 years (SD = 9.41), ranging from 21 to 60. Of the total sample, 92.1% (*n* = 256) were women and 7.9% (*n* = 22) men, with mean ages of 41.18 (SD = 9.45) and 37.45 (SD = 8.42), respectively. Their marital status was 25.5% (*n* = 71) single, 60.4% (*n* = 168) married, 13.7% (*n* = 38) divorced or separated, and 0.4% (*n* = 1) widowed.

### 2.2. Instruments

An ad hoc questionnaire was drafted to collect the sociodemographic data (age, sex, and marital status) and for information on profession and employment situation, including years of experience, employment situation (permanent or temporary), work shifts (rotating, 23 or more hours, nights only, and morning/afternoon), and number of patients attended to in a work day. Four different surveys were administered. 

The Brief Burnout Survey (CBB) [11] consists of 21 items rated on a five-point Likert-type scale that evaluates antecedents, elements, and consequences of the syndrome. Its purpose is to acquire a global assessment of burnout, and its antecedents and consequences, coinciding with the three blocks into which the questionnaire is organized.

The Brief Inventory of Emotional Intelligence for Adults (EQ-i-20M) [29] is an adaptation of the Emotional Intelligence Inventory: Young Version (EQ-i-YV) [30], validated and scaled by the authors for an adult Spanish population. It consists of 20 items with four answer choices on a Likert type scale. The EQ-i-20M is structured into five factors: Intrapersonal, Interpersonal, Stress management, Adaptability, and Mood.

The Brief Questionnaire on Perceived Social Support (CASPE) [31] was developed to study the effect of social support on health, quality of life, and general satisfaction. It consists of nine items (eight with a four-point Likert type response and another with a yes/no answer). The CASPE evaluates quantitative and qualitative aspects of family, friend, and partner relationships. The possible scores range from 9 to 35 points, where the higher the score, the greater the perceived social support. The authors found a Cronbach’s alpha reliability of 0.65 for the scale in a geriatric population. In this study, the alpha was 0.81.

The General Self-Efficacy Scale [32] consists of 10 items in a four-point Likert-type response format that evaluates a person’s perception of own personal competence in effectively managing different stressful situations. Sanjuán et al. [33] analyzed the reliability of the scale, finding a Cronbach’s alpha coefficient of 0.87. In this study, the alpha for the internal consistency of the scale was 0.93.

### 2.3. Procedure

Before collecting the data, the participants were guaranteed compliance with the standards of information confidentiality and ethics in data processing. The questionnaires were selected in accordance with the previous literature on burnout and were administered on an online Web platform. To control random answers or incongruences, a series of control questions were included for their detection, and such cases were then discarded from the study sample. The study was approved by the Bioethics Committee of the University of Almeria (Spain).

### 2.4. Data Analysis

First, correlation analyses were performed to explore the relationships between the quantitative variables and Student’s t and analyses of variance (ANOVA) were completed for the categorical variables. Then, a binary logistic regression was completed using the Enter method. The dependent variable (burnout) was dichotomized considering our proposal for the diagnosis of burnout, with a cutoff at 25 points. Thus, a person who scored over 25 points was considered affected by the syndrome [11]. The predictor variables used were sex, employment situation (permanent or temporary), number of patients attended to during a workday, emotional intelligence (intrapersonal, interpersonal, stress management, adaptability, and mood), general self-efficacy, and perceived social support. Originally, variables such as age, years of work experience, and type of shift worked (rotating, 24 h, nights only, morning/afternoon) were also included. In this case, dummy variables were created because this variable was a polytomous categorical variable. These two variables, along with the above, were proposed as possible predictors of burnout in a logistic regression using the forward Wald method, which excluded them from the model. Finally, a nonlinear predictive Chi-square Automatic Interaction Detector (CHAID) regression and classification tree were constructed. All analyses were performed using SPSS v. 23.0 statistical software for Windows.

## 3. Results

### 3.1. Burnout, Sociodemographic Variables, and Job Characteristics

First, a correlation analysis was used to check the relationships between the burnout scores and the continuous quantitative variables. A negative correlation was observed between burnout and age (*r*= −0.24; *p* < 0.001). Conversely, no correlations with burnout were found for either the number of patients attended to during the workday (*r* = 0.10; *p* = 0.07) or years of work experience (*r* = −0.05; *p* = 0.35).

Another variable related to the work context originally considered was the type of work shift (rotating, 24 h, nights only, or morning/afternoon), but when the ANOVA was applied, no statistically significant differences in the groups were found (*F* = 0.85; *p* = 0.46). On the contrary, for employment situation, the group of professionals with a permanent contract (*M* = 21.38; *SD* = 6.31) showed a significantly higher mean score in burnout (*t* = −3.30; *p* < 0.01), than those with a temporary contract (*M* = 18.87; *SD* = 5.45).

Finally, no statistically significant differences in burnout scores (*t*= −1.48; *p* = 0.13) were found between men (*M* = 17.82; *SD* = 4.07) and women (*M* = 19.73; *SD* = 5.91).

### 3.2. Burnout Relationships with Emotional Intelligence, Self-Efficacy, and Perceived Social Support Variables

As shown in Table 1, the Burnout Syndrome score is significantly related negatively with all the emotional intelligence factors (Intrapersonal: *r*= −0.26; *p* < 0.001; Interpersonal: *r* = −0.29; *p* < 0.001; Adaptability: *r* = −0.34; *p* < 0.001; Mood: *r* = −0.41; *p* < 0.001; and Stress management: *r*= −0.32; *p* < 0.001) (Table 1).

In addition, both self-efficacy (*r* = −0.37; *p* < 0.001) and perceived social support (*r* = −0.20; *p* < 0.01) had significant negative correlations with burnout.

### 3.3. Logistic Regression Model

For the logistic regression analysis with the burnout syndrome as the dependent variable, it was previously dichotomized into two categories, participants affected by the syndrome, representing 16.2% (*n* = 45), and those not affected, at 83.8% (*n* = 233).

The predictor variables entered in the equation were sex, employment situation, patients attended to, self-efficacy, perceived social support, and the five emotional intelligence factors: intrapersonal, interpersonal, stress management, adaptability, and mood. Table 2 shows these variables, the regression coefficients, the standard error of estimation, and the Wald statistic, with degrees of freedom and associated probability, the coefficient of partial correlation, and the cross-product ratio.

The odds ratio or cross-product ratio found for each variable showed that: (1) the risk of burnout is higher in younger professionals and those with a permanent employment situation, and (2) the level of general perceived self-efficacy acts as a protection factor insofar as the likelihood of having burnout. Thus, subjects with higher mean scores in this construct have a lower risk of developing the syndrome. (3) Of the emotional intelligence elements, stress management is the factor significantly involved in the logistic equation, implying a protective effect.

The overall model fit (*χ^2^*= 69.64; degrees of freedom (df) = 10; *p* < 0.001) was confirmed by the Hosmer-Lemeshow test (*χ^2^*= 7.77; gl = 8; *p* = 0.45). Moreover, the Nagelkerke *R^2^* showed that 38.1% of the variance in the response variable was explained by the logistic regression model. Similarly, in the case classification table, the likelihood of the logistic function being right was 86.1%, with a false positive rate of 0.03 and false negative rate of 0.33 (Table 2).

**Figure 1:**
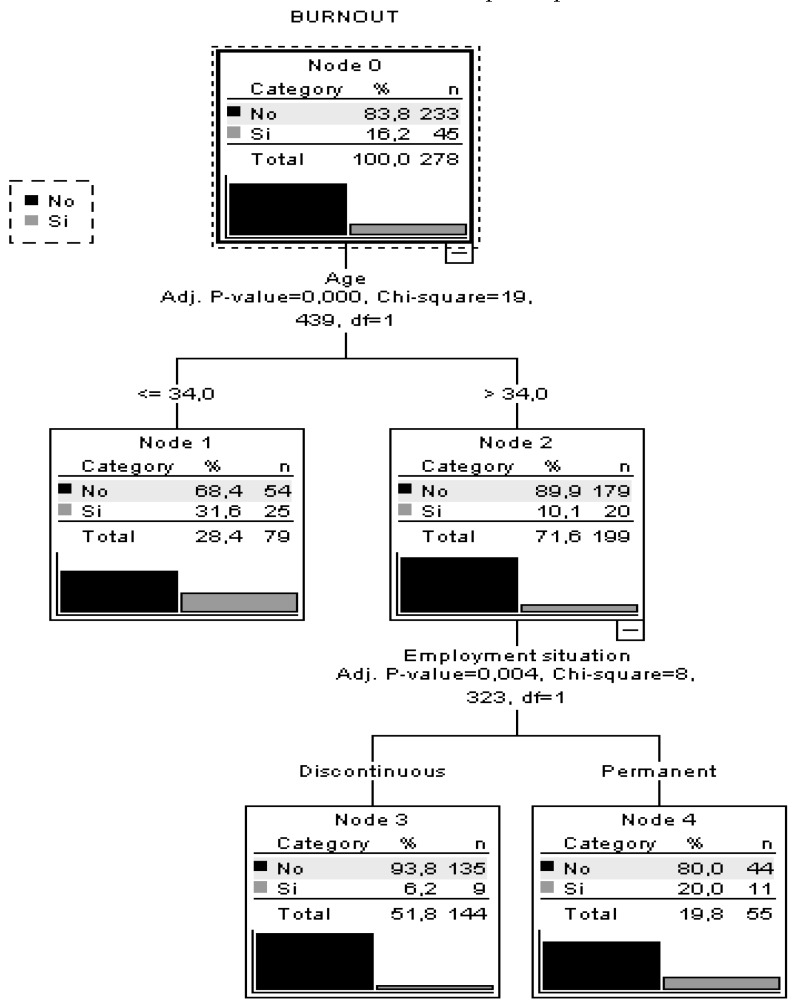
Regression and classification tree burnout.

As observed in the decision tree (Figure 1), age is the best predictor of burnout. Participants under 34 years old had the highest risk of burnout (31.6%). The lowest risk of burnout (93.8%) was found for those over 34 years and with discontinuous work. The goodness of fit of model functioning can be observed in its correct classification of 83.8% of the participants.

## 4. Discussion

Burnout in employees in the healthcare sector has awakened increasing scientific interest since its study began [7,8]. However, the volume of empirical studies on Certified Nursing Aides (CNAs) is smaller than for other workers in the healthcare field [17]. This difference may be due to a lack of academic attention or the consideration that the job conditions and duties of CNAs are less demanding than in other healthcare professionals and employees, and therefore, less vulnerable to development of this syndrome.

In this study, the prevalence of burnout in CNAs was lower than in empirical studies found in the review of the literature [4]. This may have been a consequence of the differences in job contexts where CNAs perform their duties, as many more studies have been completed on homecare than in hospital contexts [18].

The data in our study showed that Emotional Intelligence is especially important in occupational fields that require strong social interaction, acting as an important protective factor for burnout, and related significantly and positively to job performance, job motivation, and client satisfaction. In fact, persons with high emotional instability have been demonstrated to be more prone to burnout symptoms [27]. Workers with inefficient coping strategies for job stress and who experience the feeling of having little control of the situation also are more likely to feel ineffective in their work, and therefore have a higher risk of burnout [14].

These results confirm the Job Demands-Resources Model [6], where employee personal resources such as Emotional Intelligence and perceived Self-Efficacy buffer the negative impacts of job demands and are antecedents of Engagement and Job Performance [12]. Perceived Social Support would also be a job resource of relevance in preventing the development of negative attitudes acting as a buffer between job demands and burnout, as would Feedback and Coaching by the supervisor [6].

The results of our sample characteristics are congruent with previous studies, as employees with permanent contracts showed higher levels of emotional exhaustion than those with a temporary contract [23]. On the contrary, the data did not confirm that work shifts, overwork, or time in the job had any significant relationship with CNA burnout scores. However, previous studies have shown that employees with permanent contracts and longer time in the job usually show burnout symptoms, which may be due to routine and monotony [23].

Data acquired on the sociodemographic variables confirmed the results of previous studies. An inverse relationship was found between age and burnout, suggesting that younger people have less work experience, and therefore, fewer strategies for coping with job stress in the healthcare setting [24,25]. Nevertheless, unlike other studies that have described women as having a higher risk of developing burnout [19], no significant differences were found between men and women [2,4].

The results of this study have important practical implications. As perceived social support was considered a protection factor, as were employee emotional intelligence and perceived self-efficacy, organizations should promote training programs in coaching and transformational leadership to promote the wellbeing and organizational commitment of the CNAs.

However, our results must be considered with precaution due to the following limitations. First, the data were acquired from online questionnaires filled out by the employees and could show biases. Second, as the sample used is very specific, the results may not be generalized to the whole healthcare environment. Third, the study design was not able to determine whether burnout scores remained constant over time. Finally, in Spain, CNA is a profession of mostly women, which may be reflected in our sample and may limit the results.

In spite of these limitations, future studies may advance this line of research. The set of variables used in this study should be widened to include aspects related to demands (e.g., the role of ambiguity, stressful events, role conflict, etc.) and resources (e.g., leadership, autonomy, etc.) as well as engagement and performance to complete the Job Demands-Resources Model and provide better understanding of burnout in CNAs.

## 5. Conclusions

Burnout in Certified Nursing Aides is allied with organizational, personal and sociodemographic factors.

The risk of burnout is higher in younger persons and in permanently employed professionals. 

General self-efficacy and stress management act as protective factors against the likelihood of burnout.

These results confirm the Job Demands-Resources Model.

## Figures and Tables

**Table 1:** Correlations between burnout and emotional intelligence, self-efficacy, and social support variables.

		EQ-i-20M					EAG	CASPE
		Intrapersonal	Interpersonal	Stress Management	Adaptability	Mood	Self-Efficacy	Social Support
CBB	Burnout	−0.26 ***	−0.29 ***	−0.32 ***	−0.34 ***	−0.41 ***	−0.37 ***	−0.20 **
EQ-i-20M	Intrapersonal	1	0.58 ***	0.13 *	0.54 ***	0.49 ***	0.45 ***	0.43 ***
	Interpersonal		1	0.11	0.68 ***	0.55 ***	0.55 ***	0.51 ***
	Stress management			1	0.15 **	0.22 ***	0.16 **	0.08
	Adaptability				1	0.69 ***	0.70 ***	0.44 ***
	Mood					1	0.66 ***	0.43 ***
EAG	Self-efficacy						1	0.45 ***

Note: * The correlation is significant at 0.05; ** The correlation is significant at 0.01; *** The correlation is significant at 0.001.

**Table 2:** Results derived from the logistic regression for probability of burnout.

Variables	β	S.E.	Wald	df	Sig.	Exp(β)	CI 95%
Age	−0.064	0.023	7.692	1	0.006	0.938	0.897–0.981
Employment situation _(Permanent)_	1.137	0.404	7.899	1	0.005	3.116	1.411–6.885
Users attended to	0.001	0.004	0.027	1	0.870	1.001	0.992–1.009
General self-efficacy	−0.123	0.056	4.838	1	0.028	0.884	0.792–0.987
Perceived social support	0.038	0.071	0.286	1	0.593	1.038	0.904–1.192
Intrapersonal	−0.132	0.081	2.669	1	0.102	0.876	0.748–1.027
Interpersonal	−0.036	0.138	0.066	1	0.797	0.965	0.736–1.265
Stress management	−0.275	0.110	6.259	1	0.012	0.759	0.612–0.942
Adaptability	0.280	0.171	2.666	1	0.103	1.323	0.945–1.851
Mood	−0.215	0.130	2.746	1	0.097	0.807	0.626–1.040
Constant	2.672	1.798	2.210	1	0.137	14.474	
